# Transforming PrEP marketing: understanding the place of PrEP in the hearts and minds of adolescent girls and young women in sub‐Saharan Africa

**DOI:** 10.1002/jia2.26480

**Published:** 2025-07-02

**Authors:** Emily de Lacy Donaldson, Elmari Briedenhann, Patriciah Jeckonia, Casey Bishopp, Anelde Greeff, Definate Nhamo

**Affiliations:** ^1^ FHI 360 Durham North Carolina USA; ^2^ Wits RHI Johannesburg South Africa; ^3^ LVCT Health Nairobi Kenya; ^4^ 2Stories Cape Town South Africa; ^5^ Pangaea Zimbabwe Harare Zimbabwe

**Keywords:** adolescent, communication, demand generation, female, HIV prevention, marketing

## Abstract

**Introduction:**

Adolescent girls and young women (AGYW) in sub‐Saharan Africa (SSA) remain one of the populations most affected by HIV. As HIV prevention options expand—such as the introduction of the dapivirine ring, long‐acting injectable cabotegravir and other potential long‐acting methods—alongside oral pre‐exposure prophylaxis (PrEP), AGYW will have a choice of PrEP methods for HIV prevention, referred to as the PrEP category. Marketing and demand generation must evolve to communicate this choice to AGYW in real‐world settings across SSA.

**Methods:**

Using a phased approach to learn, build, iterate and validate, we developed a brand positioning strategy for the PrEP category for AGYW. In 2022, a review of existing and historic oral PrEP campaigns informed initial insights (learn). In 2023, these were further explored and developed with eight young women representatives under the age of 30 (build), then refined with PrEP implementers and Ministry of Health representatives from eight SSA countries (iterate), through five consultative virtual workshops of up to 25 participants each. Insights were funnelled through a private sector marketing framework—the 4C's—to develop a single key brand benefit (KBB), ensuring it was culturally relevant, category‐specific, consumer‐driven and product (company)‐true. The KBB was then creatively applied to posters, narratives and key messages for validation with AGYW (validate). From July to August 2023, 121 AGYW aged 18–24 participated in 16 group discussions to validate the brand positioning strategy; 44 in South Africa (6 groups), 32 in Zimbabwe (4 groups) and 45 in Kenya (6 groups).

**Results:**

Post‐validation, an optimized KBB emerged: PrEP affirms that self‐love is strength—positioning PrEP as a way for AGYW to prioritize their physical health and mental wellbeing, and live a life uninterrupted by HIV. We developed a deeper understanding of the influences shaping AGYW's relationship with the PrEP category, answering: *What do AGYW feel in their hearts and think in their minds about PrEP?*

**Conclusions:**

This strategic, evidence‐informed brand positioning—developed with AGYW, confirms that communication to promote PrEP uptake and continued use must resonate with AGYW's inner strength and frame PrEP use as an act of self‐love. It offers a powerful foundation for clear, consistent and inspiring communication that engage and retain AGYW's attention.

## 1 INTRODUCTION

In sub‐Saharan Africa (SSA), women and girls account for 61% of all HIV acquisitions that occur in people older than 15, and adolescent girls and young women (AGYW) are still the most affected [1], therefore, making them a critical audience for HIV pre‐exposure prophylaxis (PrEP), both in terms of promoting uptake and continued use [2, 3, 4]. The introduction of the dapivirine ring (PrEP ring) and long‐acting injectable cabotegravir (CAB PrEP) alongside oral PrEP means that AGYW in many settings will have a choice of different PrEP methods for HIV prevention, referred to as the PrEP category. Maximizing PrEP's public health impact requires effectively communicating choice in a way that resonates with AGYW.

The Maximizing Options to Advance Informed Choice for HIV Prevention (MOSAIC) consortium, an initiative funded by PEPFAR through USAID, worked to establish brand positioning for the PrEP category for AGYW. Brand positioning [5] is the foundational step in effectively communicating a choice of PrEP products to end users. It aligns all communication under a common strategy informed by understanding the audience and their needs, thereby creating specific associations and beliefs about a new product, category or service [6]. This work aims to create a regional PrEP brand for AGYW, providing an efficient starting point for tailored marketing campaign execution in SSA settings.

In September 2015, the World Health Organization approved oral PrEP for HIV prevention [7]. By mid‐2016, many SSA countries had made significant progress in introducing this breakthrough product [8]. The rapid rollout required an accelerated approach to marketing and communication, this work, however, explains the process for developing a brand positioning for the PrEP category while offering recommendations for marketing PrEP choice to AGYW.

## 2 METHODS

Brand positioning (or positioning direction) relates to the strategic approach that is applied to shape the perception of a product/service among audiences, this approach allows organizations to establish the key brand benefit (KBB) of its product/service that drives such perception [9]. The process of creating a brand positioning for the PrEP category included four phases: (1) reviewing existing audience insights and oral PrEP campaigns (learn); (2) co‐designing the KBB alongside AGYW representatives (build); (3) refining the KBB with key stakeholders (iterate); and (4) validating the final KBB with AGYW representing potential end‐users of the PrEP category (validate).

**Figure 1:**
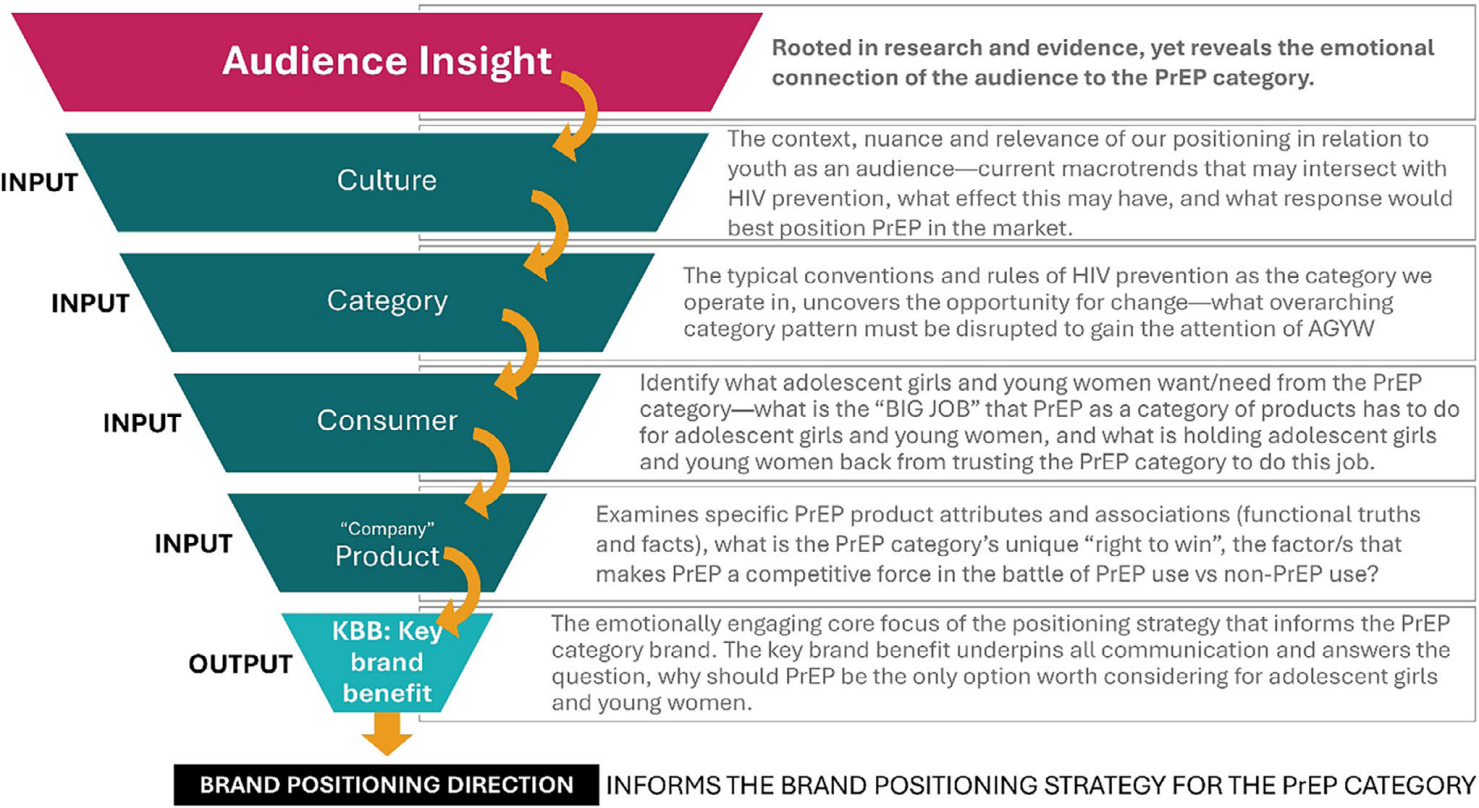
**Brand positioning funnel (4 C's) for the PrEP category**. Abbreviations: 4 C's, Culture, Category, Consumer, Company (or Product); AGYW, adolescent girls and young women; KBB, key brand benefit; PrEP Category, choice of PrEP products; PrEP, pre‐exposure prophylaxis.

MOSAIC's brand positioning for PrEP as a category of products was developed by adapting a brand‐building concept [10] from the private sector referred to as the “4C's” (company, category, consumer and culture) of marketing. We determined the direction of the positioning for PrEP as a category of products by “funnelling” audience insights gleaned from a review of existing audience insights and oral PrEP campaigns through four inputs (4C's)—culture, category, consumer and product (company)—to arrive at an output, the KBB (Figure 1). The question we sought to answer was, what do AGYW feel in their hearts and think in their minds about PrEP, and what do we, as public health programmers and implementers, want AGYW to feel and think about PrEP, and how can these two questions align to ensure communication and marketing promoting the PrEP category, is relevant and resonant to AGYW in SSA? [11]

### 2.1 Review of existing audience insights: Learn

From May to July 2022, we interviewed five programme implementers from Kenya, Lesotho, South Africa, Uganda and Zimbabwe, and held discussions with four global demand generation stakeholders to identify oral PrEP campaigns targeting AGYW. Throughout 2022, we reviewed campaign materials to assess whether they were grounded in behaviour change objectives tailored to specific audiences, recognizing that the perceived value of PrEP varies, and whether they were driven by an insight that inspires new ways of thinking and feeling. Concurrently, we reviewed a summary of AGYW and oral PrEP audience insights [12] collated under the Collaboration for HIV Prevention Options to Control the Epidemic (CHOICE) project. Audience insights reflect the perceptions, motivations and needs related to a specific behaviour, product or service [13].

Eight campaigns were identified; three focused specifically on AGYW and oral PrEP, and three themes advanced to co‐design: (1) PrEP empowers you to take control of your health; (2) PrEP enables you to be part of something that matters; and (3) PrEP helps you protect yourself from HIV.

### 2.2 Co‐design of positioning direction: Build

Themes and audience insights were presented to the MOSAIC NextGen Squad (NGS), a group of 10 young women under 30 who serve as youth advisors representing Eswatini, Kenya, Lesotho, Nigeria, South Africa, Uganda, Zambia and Zimbabwe [14]. Eight NGS members, five of whom were under 24, were engaged for ongoing input to ensure the direction brought into testing was resonant, relevant and adaptable to a rapidly changing youth culture.

Through a series of three virtual workshops, eight NGS members investigated existing insights to select and refine one that reflects today's AGYW by participating in emotive imagery exercises and in‐depth discussions. The NGS helped focus on insights that are most likely to encourage and inspire positive action among AGYW by applying a gender‐transformative lens [15] to ensure the insight selected for the brand positioning strategy supports equity; promotes the relative position of women, girls and marginalized groups; and does not reinforce gender inequalities or stereotypes.

The eight NGS members participated in virtual workshops and voted for four audience insights for developing draft KBBs. We analysed workshop notes to understand the emotional connections, perceptions and values linked to each insight, and used these to draft KBBs for review. In a final workshop, these eight NGS members live edited the drafts. Differences across countries were addressed by aligning around commonalities.

### 2.3 Refinement with key stakeholders: Iterate

Brand positioning typically involves a narrow set of stakeholders; however, this work intentionally engaged many stakeholders to ensure buy‐in. On average, 20–25 programme implementers and National Ministry of Health (MOH) officials representing Eswatini, Kenya, Lesotho, Nigeria, South Africa, Uganda, Zambia and Zimbabwe attended a virtual workshop (five workshops in total were held to accommodate scheduling conflicts) where stakeholders reviewed and provided input into the draft KBBs, these virtual workshops were co‐facilitated by on average five NGS representatives and the authors. Ongoing engagement with MOH representatives allowed for alignment with country priorities and contexts.

Given the range of geographies of stakeholders attending the virtual workshops, feedback was analysed for commonalities across settings, including specific cultural trends, concerns and excitement for the PrEP category, and effective marketing practices in different settings, while providing their preferences for a final KBB for validation. Stakeholder feedback allowed the authors to further refine the positioning direction to be as relevant and resonant to AGYW in SSA settings.

### 2.4 Validation with AGYW: Validate

Before validation discussions with AGYW, FHI 360's Institutional Review Board reviewed and considered the activity as non‐research. Three creative interpretations of the KBB, called “territories,” were developed—each with posters and narratives. All participants provided written consent. Between June and August 2023, 121 AGYW aged 18–24 participated in 16 group discussions: 44 in South Africa (6 groups), 32 in Zimbabwe (4 groups) and 45 in Kenya (6 groups). AGYW engaged in exercises exploring each territory across metrics such as value proposition, functional benefit, trust, clarity, relevance, emotional resonance, and memorability and motivation.

Each discussion session followed a consistent format. AGYW engaged in a short “get to know you” activity to introduce themselves, received basic information about the different PrEP methods, followed by selecting words from a word cloud that best represented their feelings about PrEP. The three creative territories were then shared, and participants rated them using a 5‐point Likert scale. Participants compared and ranked the territories based on how compelling they were, how well they communicated HIV prevention, which they were more likely to share with a friend (net promoter score) and their overall favourite. Participants also gave feedback on posters using colour‐coded notes—green (likes), red (dislikes) and yellow (suggested changes). Throughout, participants explained their ratings and choices. Each discussion had two note‐takers, and once completed, notes were systematically reviewed by a trained market researcher. Key themes were identified and aligned with the framework in Figure 1—culture, category, consumer and product—to refine the final KBB.

## 3 RESULTS

The original audience insight tells the story of PrEP and AGYW in SSA: *Life is a balancing act of what I want, what I need, and what's expected of me. Prioritising myself by taking PrEP requires that I overcome the many challenges that surround PrEP use, especially judgment and stigma. This is difficult, but I know I'm at my best when I feel good about myself because I've taken PrEP. When I feel confident and strong, my actions, decisions, and words reflect it*.

Translated into a draft KBB for validation, it aimed to capture what AGYW hope to achieve by choosing PrEP: *Strong women understand the need for kindness, compassion, and taking care of ourselves first so that we can stand strong for our families, in our communities, and in the world. PrEP is an affirmation of this strength—being strong requires being soft to yourself. PrEP is an affirmation that soft is strong*.

**Figure 2:**
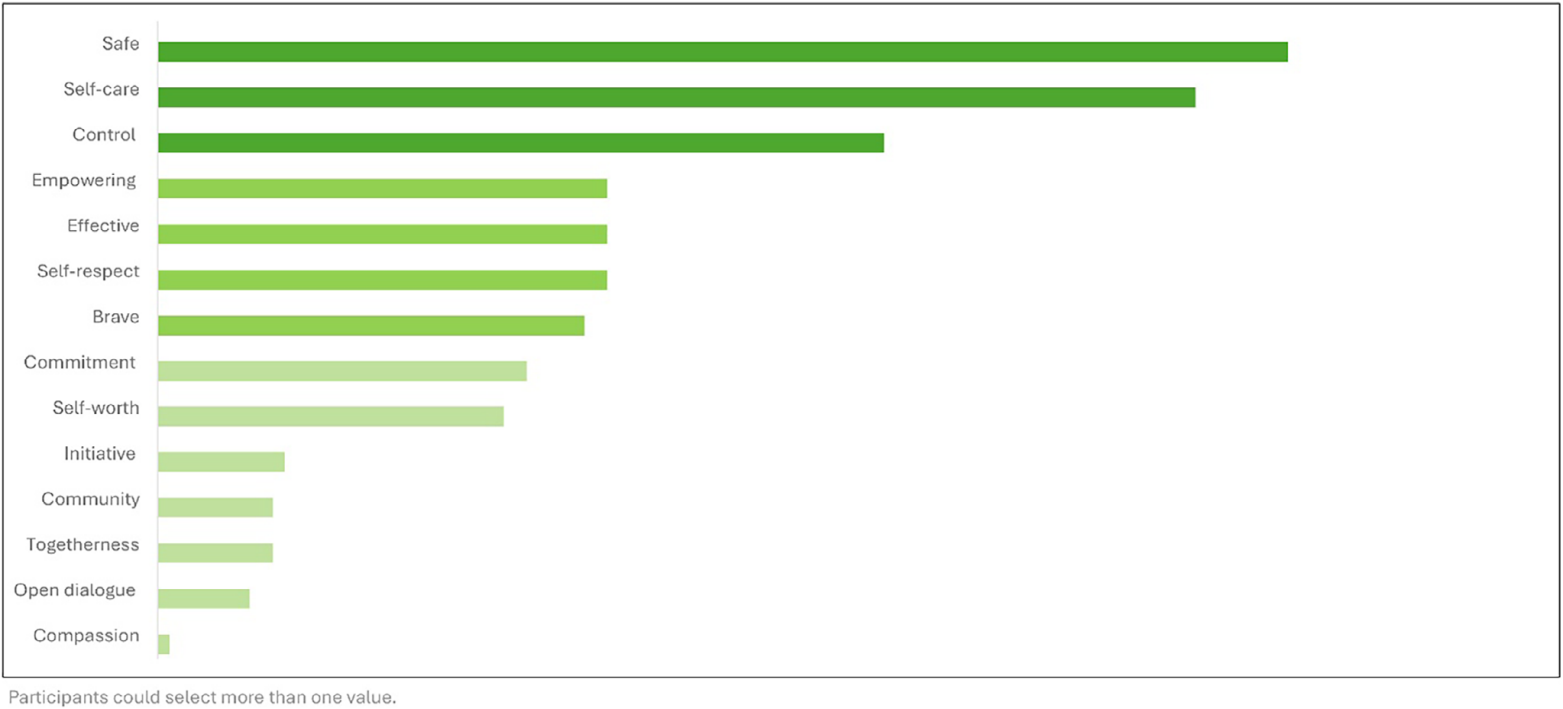
**Values AGYW associated with PrEP**. Abbreviations: AGYW, adolescent girls and young women; PrEP, pre‐exposure prophylaxis.

**Figure 3:**
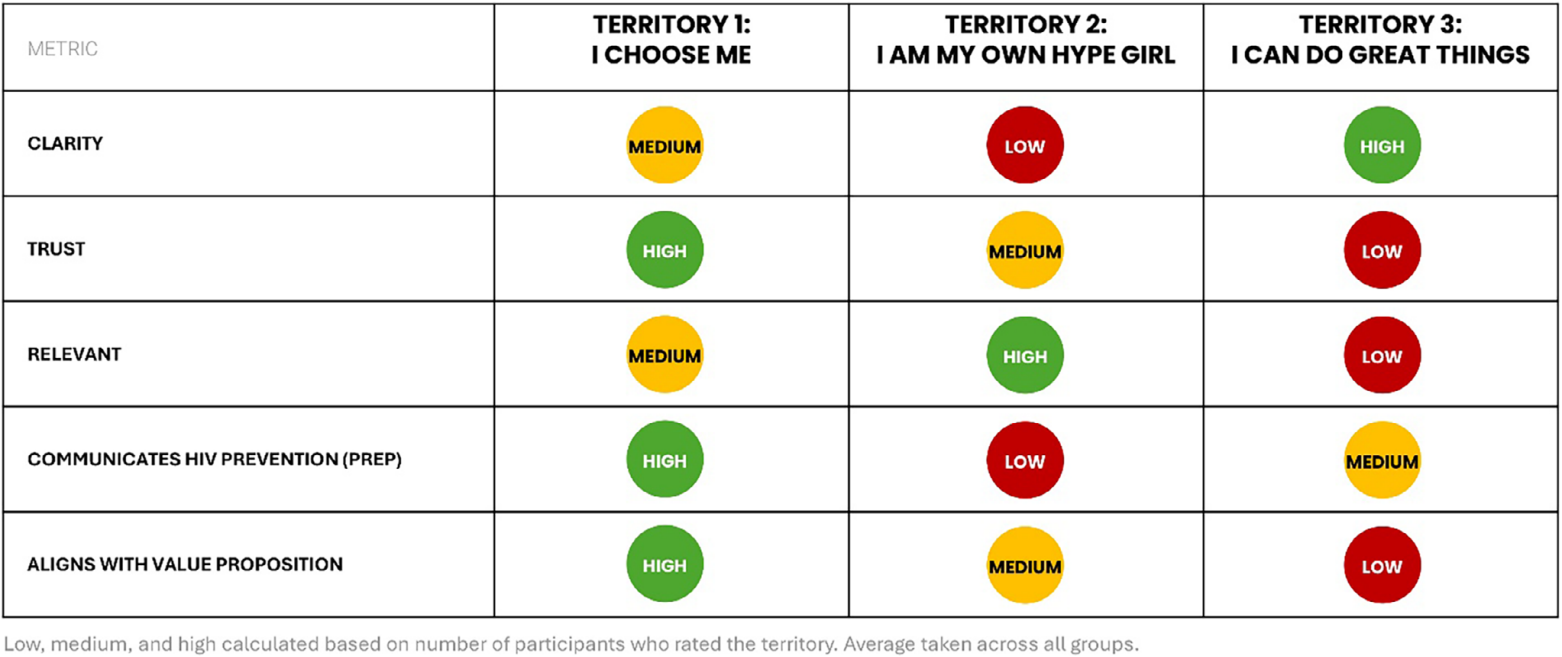
**Territory ratings from validation with AGYW**. Abbreviations: AGYW, adolescent girls and young women; PrEP, pre‐exposure prophylaxis; Territories, creative interpretations of the key brand benefit proposed for PrEP as a category of products (different PrEP products to choose from).

During validation, AGYW connected PrEP most to the values of safety, self‐care and control (Figure 2). Across the ratings, Territory 1 (I CHOOSE ME) led in clarity, trust, and communicating PrEP and aligned best with the values of safety and self‐care (Figure 3).

From the validation, the following emerged in relation to the 4 C's regarding AGYW's connection with the PrEP category:

1. Culture: PrEP confirms the strength, sense of self and power AGYW already have and supports them in putting themselves first. Today's AGYW are not seeking permission to live up to their potential. They seek programmes, services and products that affirm their self‐belief.
2. Category: AGYW strongly associate PrEP with self‐care. The concept is understood as having the confidence, self‐worth and self‐respect to care for themselves and their health. As an AGYW in Nairobi, Kenya said, “With PrEP, I'm able to know my worth. I can walk with my head high because I'm preventing HIV. It goes hand‐in‐hand with self‐care.”
3. Consumer: PrEP provides peace of mind. PrEP is seen by AGYW as an essential way to negate the anxiety of momentary perceptions of risk (e.g. after condomless sex). An AGYW in Johannesburg, South Africa, noted, “I cannot control what's happening on someone else's end, but I can control what's happening on my end. As a young person, you get a lot of pressure and can't always say, ‘I want to use a condom’. PrEP enables you to confront your feelings honestly, your invisible therapy.”
4. Product: PrEP choice gives AGYW control. AGYW acknowledge many factors outside of their control (HIV status of a partner or instances of sexual assault). PrEP offers AGYW some autonomy over their bodies. In the context of choice, there is an even greater measure of control—they can choose whether to use PrEP and which method.

Evaluation of the three territories revealed that AGYW see choosing to take PrEP as a form of self‐care. Softness as used in the original KBB was absent, feelings and beliefs about safety, control and strength emerged. This critical shift changed the KBB to reflect participants’ emotional connection with PrEP: *PrEP is a way for AGYW to prioritise their physical health and mental well‐being, to live a life uninterrupted by HIV. It affirms that self‐love is strength*. For the PrEP category to stand out as a strong brand, it should resonate with AGYW's inner strength and unwavering determination to live a full and healthy life.

## 4 DISCUSSION

Youth culture is rapidly evolving, and AGYW today, have different lived experiences from when oral PrEP campaigns were first launched in SSA. This work explored how AGYW now perceive PrEP choice, and how best to communicate PrEP to them. We found a shift in how AGYW emotionally connect with PrEP, differing from earlier campaigns. South Africa's We Are the Generation campaign [16] focused on community and purpose, Kenya's Chagua PrEP [17] framed PrEP as empowering AGYW to take control of their health and Zimbabwe's #GarawaPrEPa campaign [18] emphasized supporting AGYW to prevent HIV. While themes of control and prevention held true, today's AGYW rejected the idea that empowerment must come from external sources. Instead, self‐care emerged as the strongest emotional driver; representing self‐worth, self‐respect and inner strength. Further noting that not all audience insights should be translated into marketing. For example, framing AGYW's PrEP use primarily within intimate relationships may reinforce limiting norms by portraying these partnerships as single‐partner, heterosexual and male‐dominated [19, 20, 21, 22]. Applying a gender transformative lens [20] allows for a direction that encourages critical consciousness and situates AGYW as the most important actors in their own lives.

## 5 CONCLUSIONS

MOSAIC's brand positioning strategy [23] applied private sector marketing principles to determine how to generate demand for PrEP among AGYW for public health impact. It is intended for use as a guide by implementers developing national communications strategies, demand generation campaigns and other marketing materials. As we enter the era of PrEP choice, it is increasingly important to ensure sustained investment in evidence‐driven, audience‐centred communication that promotes uptake and continued use of PrEP products.

## COMPETING INTERESTS

The authors declare no competing interests.

## AUTHORS’ CONTRIBUTIONS

ELD and EB conceptualized the paper. ELD wrote the first draft of the paper, EB and CB contributed to editing the paper, and PJ, DN and AG reviewed and commented on drafts. EB addressed all final reviewer comments and wrote the final draft of the paper.

## FUNDING

The commentary reports on work undertaken through the MOSAIC Project. MOSAIC is made possible by the generous support of the American people through the PEPFAR and USAID.

## DISCLAIMER

The contents of this commentary are the responsibility of Maximizing Options to Advance Informed Choice for HIV Prevention (MOSAIC) and do not necessarily reflect the views of PEPFAR, USAID or the U.S. Government. MOSAIC is a global cooperative agreement (#7200AA21CA00011) led by FHI 360, with core partners Wits RHI, Pangaea Zimbabwe, LVCT Health, Jhpiego and AVAC.

## Data Availability

Where parties are interested in access to the data, please direct requests to corresponding author: ebriedenhann@wrhi.ac.za. The full brand positioning report is available at: https://www.prepwatch.org/resources/insights-report-communicating-the-prep-category-to-agyw/
